# Supplementary material for: Proteome dynamics reveal Leiomodin 1 as a key regulator of myogenic differentiation
Source: eLife. 2026 Aug 4;14:RP104331. doi: 10.7554/eLife.104331 (PMC13436964; doi:10.7554/eLife.104331)
Supplement: Peer review history

# Editor report: eLife Assessment

**DOI:** 10.7554/eLife.104331.3.sa0

**Authors:** Johnny Kim

**Affiliations:** TRON Germany

**Keywords:** Solid, Valuable

This study provides **valuable** insights into the regulation of myogenic differentiation by identifying Leiomodin 1 as a modulator of proteome dynamics during myogenic differentiation. The combination of quantitative proteomics with functional perturbation experiments offers **solid** evidence supporting the idea that SIRT1 influences perturbations of myogenic differentiation upon LMOD1 inactivation. These findings advance our understanding of muscle differentiation and will be of interest to researchers studying muscle development and related pathologies

# Referee report: Reviewer #1 (Public review)

**DOI:** 10.7554/eLife.104331.3.sa1

The main significance of this work is characterizing the function of a new gene Lmod1 in muscle stem cell biology. The study suggests an intriguing regulatory mechanism by which Sirt1 sequesters Lmod1 in a specific temporal window during myogenesis.

Comments on revisions:

The authors have satisfactorily addressed my inquires. Thank you.

# Referee report: Reviewer #2 (Public review)

**DOI:** 10.7554/eLife.104331.3.sa2

Summary:

In this manuscript, the authors identify Leiomodin-1 (LMOD1) as a key regulator of early myogenic differentiation, demonstrating its interaction with SIRT1 to influence SIRT1's cellular localization and gene expression. The authors propose that LMOD1 translocates SIRT1 from the nucleus to the cytoplasm to permit the expression of myogenic differentiating genes such as MYOD or Myogenin.

Strengths:

A major strength of this work lies in the robust temporal resolution achieved through a time-course mass spectrometry analysis of in vitro muscle differentiation. This provides novel insights into the dynamic process of myogenic differentiation, often under explored in terms of temporal progression. The authors provide a strong mechanistic case for how LMOD1 exerts its role on muscle differentiation which opens avenues to modulate.

Weaknesses:

In the revised manuscript, the authors begin to translate their in vitro findings to an in vivo context by examining SIRT1 expression across a regeneration time course (Fig. 4I). They observe an increase in SIRT1 expression concomitant with LMOD1, supporting a potential role for SIRT1 in myogenic differentiation. Future studies will be required to provide deeper mechanistic insight into SIRT1 function in vivo.

Discussion:

Overall, the study emphasizes the importance of understanding the temporal dynamics of molecular players during myogenic differentiation and provides valuable proteomic data that will benefit the field. Future studies should explore whether LMOD1 modulates the nuclear-cytoplasmic shuttling of other transcription factors during muscle development and how these processes are mechanistically achieved. Investigating whether LMOD1 can be therapeutically targeted to enhance muscle regeneration in contexts such as exercise, aging, and disease will be critical for translational applications. Additionally, elucidating the interplay among LMOD1, LMOD2, and LMOD3 could uncover broader implications for actin cytoskeletal regulation in muscle biology. The authors have nicely updated their discussion.

# Referee report: Reviewer #3 (Public review)

**DOI:** 10.7554/eLife.104331.3.sa3

Summary:

In this manuscript, the investigators identified LMOD1 as one of a subset of cytoskeletal proteins that levels increase in early stages of myogenic differentiation. Lmod1 is understudied in striated muscle and in particular in myogenic differentiation. Thus, this is an important study. It is also a very thorough study, with perhaps even too much data presented. Importantly, the investigators observed that LMOD1 appears to be important for skeletal regeneration, myogenic differentiation and that it interacts with SIRT1. Both primary myoblast differentiation and skeletal muscle regeneration were studied. Rescue experiments confirmed these observations: SIRT1 can rescue perturbations of myogenic differentiation as a result of LMOD1 knockdown.

Strengths:

Particular strengths include: an important topic, the use of primary skeletal cultures, the use of both cell culture and in vivo approaches, careful biomarker analysis of primary mouse myoblast differentiation, the use of two methods to probe the function of the Lmod1/SIRT1 pathway via using depletion approaches and inhibitors, and the generation of six independent myoblast cultures. Results support their conclusions.

Weaknesses:

(1) Figure 1. Images of cells in Figure 1A are too small to be meaningful (especially in comparison to the other data presented in this figure). Perhaps make graphs smaller?

(2) Line 148 "We found LMOD2 to be the most abundant Lmod in whole skeletal muscle." This is confusing since most, if not all, prior studies have shown that Lmod3 is the predominant isoform in skeletal muscle. The two papers that are cited are incorrectly cited. Clarification to resolve this discrepancy is needed.

(3) Figure 2. Immunofluorescence (IF) panels are too small to be meaningful. Perhaps the graphs could be made smaller and more space allocated for the IF panels? This issue is apparent for just about all IF panels - they are simply too small to be meaningful. Additionally, in many of the immunofluorescence figures, the colors that were used make it difficult to discern the stained cellular structures. For example, in Figure S1, orange and purple are used - they do not stand out as well as other colors that are more commonly used.

(4) There is huge variability in many experiments presented - as such, more samples appear to be required to allow for meaningful data to be obtained. For example, Figure S2. Many experimental groups, only have 3 samples - this is highly problematic - I would estimate that 5-6 would be the minimum.

(5) Ponceau S staining is often used as a loading control in this manuscript for western blots. The area/molecular weight range actually used should be specified. Not clear why in some experiments GAPDH staining is used, in other experiments Ponceau S staining is used, and in some, both are used. In some experiments the variability of total protein loaded from lane-to-lane is disconcerting. For example, in Figure S4C there appears to be more than normal variability. Can the protein assay be redone and the samples run again?

(6) Figure S3 - Lmod3 is included in the figure but no mention of it occurs in the title of the figure and/or legend.

(7) Abstract, line 25. "overexpression accelerates and improves the formation of myotubes". This is a confusing sentence. How is it improving the formation? A little more information about how they are different than developing myotubes in normal/healthy muscle would be helpful

(8) Impossible from IF figures presented to determine where Lmod1 localizes in the myocytes. Information on its subcellular localization is important. Does it localize with Lmod2 and Lmod3 at thin filament pointed ends?

Comments on revisions:

Many comments have been adequately addressed. However, some concerns remain.

Former Concern #2. The issue with the lack of detection of LMOD3 in their muscle samples is troublesome and has not been adequately resolved in the revised manuscript. It is a fact that most, if not all, studies on Lmod3 report that it is the most abundant isoform in skeletal muscle. This issue should be discussed in the manuscript. It is recognized that a different assay was utilized in this paper. The papers that are cited continue to remain incorrect. Specifically:

Tsukada et al., reports abundance of LMOD2 in cardiac muscle, not in skeletal muscle.

Nworu et al., 2015 reports on LMOD3 in skeletal muscle.

Kiss et al.,2020. While this paper reveals an important function for Lmod2 in thin filament length regulation, it is clearly shows many examples of high expression of Lmod3 in various skeletal muscles isolated from mice.

Former Concern #3. With respect to small sample numbers. Hopefully a statistical editor is available to comment. While this reviewer is happy that other assays were used to verify their data, the problem still remains that many experimental groups only have 3 samples (with high variability).

Former Concern #3. Many immunofluorescence panels are hard to evaluate because of their small size.

# Author comment: Author response

**DOI:** 10.7554/eLife.104331.3.sa4

**Authors:** Ellen Späth, Svenja C Schüler, Ivonne Heinze, Therese Dau, Alberto Minetti, Maleen Hofmann, Katja Hönzke, Julia von Maltzahn, Alessandro Ori

**Affiliations:** Leibniz Institute on Aging - Fritz Lipmann Institute (FLI) Jena Germany; Leibniz Institute on Aging - Fritz Lipmann Institute (FLI) Jena Germany; Leibniz Institute on Aging Jena Germany; Leibniz Institute on Aging - Fritz Lipmann Institute (FLI) Jena Germany; Leibniz Institute on Aging - Fritz Lipmann Institute (FLI) Jena Germany; Leibniz Institute on Aging - Fritz Lipmann Institute (FLI) Jena Germany; Brandenburg Technische Universität Cottbus-Senftenberg Senftenberg Germany; Leibniz Institute on Aging - Fritz Lipmann Institute (FLI) Jena Germany; Leibniz Institute on Aging - Fritz Lipmann Institute (FLI) Jena Germany

The following is the authors’ response to the original reviews.

**eLife Assessment**

This valuable study offers insights into the role of Leiomodin-1 (LMOD1) in muscle stem cell biology, advancing our understanding of myogenic differentiation and indicating LMOD1 as a regulator of muscle regeneration, aging, and exercise adaptation. The integration of in vitro and in vivo approaches, complemented by proteomic and imaging methodologies, is solid. However, certain aspects require further attention to improve the clarity, impact, and overall significance of the work, particularly in substantiating the in vivo relevance. This work will provide a starting point that will be of value to medical biologists and biochemists working on LMOD and its variants in muscle biology.

Thank you for the positive feedback on our manuscript and the constructive criticism provided by the reviewers that helped us improve our manuscript.

**Public Reviews:**

**Reviewer #1 (Public review):**

This manuscript by Ori and colleagues investigates the role of Lmod1 in muscle stem cell activation and differentiation. The study begins with a time-course mass spectrometry analysis of primary muscle stem cells, identifying Lmod1 as a pro-myogenic candidate (Figure 1). While the initial approach is robust, the subsequent characterization lacks depth and clarity. Although the data suggest that Lmod1 promotes myogenesis, the underlying mechanisms remain vague, and key experiments are missing. Please find my comments below.

We thank the reviewer for the positive feedback on our manuscript and the helpful comments, which helped improve it.

(1) The authors mainly rely on coarse and less-established readouts such as myotube length and spherical Myh-positive cells. More comprehensive and standard analyses, such as co-staining for Pax7, MyoD, and Myogenin, would allow quantification of quiescent, activated, and differentiating stem cells in knockdown and overexpression experiments. The exact stage at which Lmod1 functions (stem cell, progenitor, or post-fusion) is unclear due to the limited depth of the analysis. Performing similar experiments on cultured single EDL fibers would add valuable insights.

We thank the reviewer for this comment. In addition to performing standard measurements such as staining for Myogenin and Myosin Heavy Chain (Figure S2H), we focused on morphological readouts, such as myotube formation, because LMOD1 is an actin cytoskeleton-associated protein. Therefore, we reasoned its function would be most directly reflected in structural changes during differentiation, rather than solely in early transcriptional markers.

Regarding the use of standard markers, we have already performed co-staining for Myogenin and Myosin Heavy Chain (MHC), which effectively quantifies early myogenic committed (Myogenin+/MHC-) and terminally differentiating (Myogenin+/MHC+) cells (Figure S2H). We did not include Pax7 as our primary culture system consists of already activated myoblasts, where Pax7 is not a reliable marker of quiescence. Our data also suggest that Lmod1 is important in regulating differentiation with comparably only mild effects on proliferation (S2D-E), therefore, we focused on this stage of myogenesis.

Our focus on differentiation over activation is further supported by multiple lines of evidence. First, analysis of publicly available transcriptome datasets reveals that Lmod1 mRNA levels actually decrease upon Muscle Stem Cell (MuSC) activation, suggesting its primary role is not during this initial phase. We added this data for clarification to Figure S1B. This aligns perfectly with our in vivo data from cardiotoxin-induced muscle regeneration, where abundance of LMOD1 protein peaks at days 4-7 post-injury — a time point coinciding with new myofiber formation and maturation — rather than during the initial activation and proliferation phase (days 1-3) (Figure 4I).

Given this strong evidence pointing to a primary role for LMOD1 during the later stages of differentiation, we believe our current analyses are the most relevant. While single EDL fiber cultures are valuable for studying the quiescence-to-activation transition, they would not provide significant additional insight into the specific differentiation-centric mechanism we are investigating here. We are confident that our chosen readouts appropriately address Lmod1's function in the differentiation of myoblasts and formation of myotubes.

(2) In supplementary Figure 2E, the distinction between Hoechst-positive cells and total cell counts is unclear. The authors should clarify why Hoechst-positive cells increase and relabel "reserve cells," as the term is confusing without reading the legend.

We thank the reviewer for pointing out the confusion regarding the naming of the cell populations and the increase in Hoechst-positive cells. We have now modified this and revised the terminology used in Figure S2E to improve clarity. Specifically, we have relabeled "reserve cells" as "non-proliferating myoblasts (Ki67-/Hoechst+)" to describe these cells more accurately without requiring the legend for interpretation. Regarding the increase in Hoechst-positive cells, we observed a slight (26%) but significant decrease in the number of proliferating myoblasts (Ki67+/Hoechst+) (Figures S2D and S2E). The relative increase in non-proliferating (Ki67-/Hoechst+) cells is a consequence of the significant reduction in the number of proliferating cells (Ki67+/Hoechst+) cells. Importantly, the total cell count (sum of Ki67-/Hoechst+) and (Ki67+/Hoechst+) remained stable. This has been clarified in the revised figure legend and main text as follows:

“This was accompanied by a proportional increase in non-proliferating myoblasts (Ki67-/Hoechst+), while the total Hoechst-positive cell count (Ki67+/Hoechst+ and Ki67-/Hoechst+) remained unchanged (Figure S2E).”

(3) The specificity of Lmod1 and Sirt1 immunostaining needs validation using siRNA-treated samples, especially as these data form the basis of the mechanistic conclusions.

We have validated the specificity of the LMOD1 antibody using multiple approaches. Specifically, we performed immunofluorescence and immunoblotting on Lmod1 siRNA-transfected samples, where we observed a significant reduction in the Lmod1 protein signal compared to control conditions (see manuscript data from Figure S2G).

Additionally, LMOD1 overexpression experiments demonstrated a corresponding increase in the signal for LMOD1 using immunofluorescence analyses, confirming the specificity of the antibody for detecting LMOD1.

For the reviewers’ interest, we add Author response image 1:

**Author response image 1.** Specificity of antibodies detecting LMOD1. Representative immunofluorescence images of LMOD1 in primary myoblast cultures following siLmod1 knockdown, LMOD1 overexpression, or controls transfected with a non-targeting siRNA (siCtrl) after one day of differentiation. LMOD1 (purple), SIRT1 (yellow), and nuclei (Hoechst, blue). Scale bar: 10 µm.

For the SIRT1 antibody used in our immunostaining, the specificity was validated by transfecting primary myoblasts with siRNA targeting Sirt1 and performing immunoblot analyses (Figure S5A). These showed a significant reduction in SIRT1 protein levels, confirming both the effectiveness of the siRNA and, critically, the antibody's ability to specifically recognize and detect SIRT1 protein. Furthermore, the same SIRT1 antibody was utilized in our nuclear-cytoplasmic fractionation experiments (Figure S4C), and its ability to detect SIRT1 in the expected subcellular compartments further supports its specific binding to SIRT1. While direct immunofluorescence on Sirt1 siRNA-transfected samples was not performed, the robust demonstration of the antibody's specificity for Sirt1 protein via immunoblotting (i.e., correct molecular weight band, significantly reduced by Sirt1 siRNA) and its distribution in subcellular fractions, which is fully consistent with the localization immunostaining performed at the same time points (compare Figure S4C and 5A), provide strong evidence on the antibody’s specificity, also in immunofluorescence experiments.

(4) The authors must test the effect of Lmod1 siRNA on Sirt1 localization, as only overexpression experiments are shown

We carefully considered performing this experiment. However, the knockdown of Lmod1 significantly impairs myogenic differentiation, a crucial cellular process that itself can influence protein localization. Consequently, if SIRT1 localization would be altered following knockdown of Lmod1, it would be challenging to disentangle whether this was a direct result of LMOD1 absence impacting SIRT1 trafficking or an indirect consequence of the cells failing to differentiate properly. This would make it difficult to draw clear conclusions regarding a direct causal link between LMOD1 and SIRT1 localization from such an experiment. Therefore, we focused on overexpression experiments, where we could demonstrate that altering LMOD1 levels is sufficient to affect SIRT1 localization. Our nuclear-cytoplasmic fractionation experiments clearly show that LMOD1 overexpression leads to changes in SIRT1 distribution (Figure 5H-K). These findings provide evidence that LMOD1 can directly modulate SIRT1 localization, supporting our mechanistic conclusions.

(5) In Figure S3, the biotin signal in LMOD2 samples appears weak. The authors need to address whether comparing LMOD1 and LMOD2 is valid given the apparent difference in reaction efficiency. It would also help to highlight where Sirt1 falls on the volcano plot in S3B.

We agree that the overall biotin signal on the streptavidin blot for the LMOD2-BirA* sample appears weaker than for LMOD1-BirA*. To provide a more direct comparison of the bait proteins themselves, we have now added a bar graph to the revised Figure S3D, which quantifies the relative abundance of LMOD1 and LMOD2 bait proteins in the pull down experiments. This analysis shows that the levels of LMOD1-BirA* and LMOD2-BirA* were comparable in our BioID samples. Furthermore, the validity of the LMOD2 BioID experiment is strongly supported by the identification of several known LMOD1 and LMOD2 interaction partners. As shown in the dataset, well-established interactors such as TMOD1, TPM3, and TMOD3 were identified, with some even showing stronger enrichment with LMOD2 than with LMOD1. This confirms that the biotinylation reaction was efficient enough to capture proximal proteins for both baits.

Regarding SIRT1, we have now highlighted in yellow its position on the volcano plot in the revised Figure S3E. As can be seen, SIRT1 was identified in the LMOD1-BirA sample and showed enrichment. We believe these clarifications, along with the additional expression data and the successful identification of known interactors, confirm the validity of our comparative BioID analysis.

(6) The immunostaining data suggest that Lmod1 remains cytoplasmic throughout differentiation, whereas Sirt1 shows transient cytoplasmic localization at day 1 of differentiation. The authors should explain why Sirt1 is not constantly sequestered if Lmod1's cytoplasmic localization is consistent. It is also unclear whether day 1 is the key time point for Lmod1 function, as its precise role during myogenesis remains ambiguous.

We thank the reviewer for this comment. We have no data explaining why SIRT1 is not constantly sequestered while LMOD1 remains consistently cytoplasmic. We can only speculate that the transient cytoplasmic localization of SIRT1 may be linked to the availability and functional role of LMOD1 throughout the differentiation process. While LMOD1 is present at low levels in proliferating primary myoblasts, its expression increases upon the initiation of differentiation (Figure 2A). Initially, during the early stages of differentiation, LMOD1 may not be required for actin nucleation as the major remodeling of the cytoskeleton has not yet begun. During this phase, LMOD1 might have the capacity to sequester SIRT1 in the cytoplasm.

However, as differentiation progresses and morphological changes take place, LMOD1 may switch its functional role to actin nucleation, thereby releasing SIRT1. This transition could explain why SIRT1 is free to localize transiently to the cytoplasm, particularly at day 1, when cytoskeletal remodeling is beginning but not yet fully established.

Additionally, as LMOD1 and SIRT1 are known to colocalize in the nucleus, they may exit the nucleus together. Once in the cytoplasm, LMOD1 may become engaged in actin nucleation, allowing SIRT1 to function independently, which could explain the transient nature of SIRT1’s cytoplasmic localization.

We have acknowledged this gap in our understanding in the discussion of the revised manuscript:

“Our immunostaining data show that while LMOD1 is consistently cytoplasmic, its partner SIRT1 is only transiently localized in the cytoplasm. This suggests that their interaction is dynamically regulated. We hypothesize that the function of LMOD1 is determined by the changing availability of its binding partners during differentiation. During the initial phase, LMOD1 may primarily function to sequester SIRT1, a key regulator of myogenic genes. As differentiation proceeds, the increased expression of cytoskeletal components, such as its canonical partners TMODs and TPMs, likely shifts the function of LMOD1 towards its role in actin nucleation. This molecular switch, potentially driven by a change in the interactome of LMOD1, could then result in the release of SIRT1 from the cytoplasm. Such a mechanism may coordinate transcriptional regulation with cytoskeletal remodeling during myoblast differentiation.”

(7) The introduction does not sufficiently establish the motivation or knowledge gap this work aims to address. Instead, it reads like a narration of disparate topics in a single paragraph. The authors should clarify the statement in line 150, "since this protein has been...,".

We thank the reviewer for requesting clarification regarding our focus on LMOD1 (Introduction and Line 150 in the original submission). In the revised manuscript, we shortened the introduction and more clearly emphasized the motivation of our study:

“Although these mechanisms contribute to remodeling the cellular architecture of MuSCs, a comprehensive understanding of the temporal dynamics of proteome remodeling during differentiation remains lacking. To address this knowledge gap, we performed an unbiased proteomic analysis of the early stages of myogenic differentiation to identify previously unrecognized proteins involved in this process and to examine how they functionally interact with established regulatory pathways.”

Our decision to focus on LMOD1 was driven by its significant upregulation in our temporal proteome dataset, together with its previously uncharacterized role in primary myoblasts. Furthermore, to strengthen the interpretation of LMOD1’s role, particularly in the context of aging, we have integrated a new analysis of published transcriptomic datasets. This can be found in the main text as follows:

“Surprisingly, we detected LMOD1 in freshly isolated muscle stem cells (MuSCs), but not LMOD2. Additionally, we observed that the protein levels of LMOD1 increased in MuSCs isolated from older mice (Figure 2C and Figure S1B). We further analyzed published transcriptomic data sets that describe changes between young and old MuSCs in both quiescent and activated states in young and old animals (Liu et al. 2013; Lukjanenko et al. 2016). In these analyzed transcriptomic data sets, Lmod1 was found to be significantly downregulated during the activation of MuSCs in both young and old mice (see Figure S1B).

To assess the in vivo relevance of our finding, we queried two proteomic datasets of freshly isolated MuSCs and four different skeletal muscles (gastrocnemius, G; soleus, S; tibialis anterior, TA; extensor digitorum longus, EDL) (Schüler et al. 2021). We found LMOD2 to be the most abundant leiomodin protein in whole skeletal muscle, consistent with data from (Tsukada et al. 2010; Nworu et al. 2015; Kiss et al. 2020), while the overall abundance of LMOD1 was lower since this protein has been mainly associated with smooth muscle cells (Nanda and Miano 2012; Conley et al. 2001; Nanda et al. 2018) (Figure 2B).”

Overall, while the identification of Lmod1 as a pro-myogenic factor is convincing, the mechanistic insights are insufficient, and the manuscript would benefit from addressing these concerns.

We thank the reviewer for their constructive criticism. In the revised manuscript, we have strengthened our mechanistic insights and the validation of our findings by implementing the suggestions of the reviewers and including new experimental data to address their concerns.

**Reviewer #2 (Public review):**

Summary:

In this manuscript, the authors identify Leiomodin-1 (LMOD1) as a key regulator of early myogenic differentiation, demonstrating its interaction with SIRT1 to influence SIRT1's cellular localization and gene expression. The authors propose that LMOD1 translocates SIRT1 from the nucleus to the cytoplasm to permit the expression of myogenic differentiation genes such as MYOD or Myogenin.

Strengths:

A major strength of this work lies in the robust temporal resolution achieved through a time-course mass spectrometry analysis of in vitro muscle differentiation. This provides novel insights into the dynamic process of myogenic differentiation, often under-explored in terms of temporal progression. The authors provide a strong mechanistic case for how LMOD1 exerts its role in muscle differentiation which opens avenues to modulate.

We thank the reviewer for the positive feedback on our manuscript and the insightful comments which helped to improve the manuscript!

Weaknesses:

One limitation of the study is the in vivo data. Although the authors do translate their findings in vivo for LMOD1 localization and expression, the cross-sectional imaging is not highly convincing. Longitudinal cuts or isolated fibers could have been more useful specimens to answer these questions. Moreover, the authors do not assess their in vitro SIRT1 findings in vivo. A few key experiments in regenerating or aged mice would strengthen the mechanistic insight of the findings.

We agree that longitudinal cuts and isolated fibers can provide excellent morphological detail for specific questions. However, for our primary objective in this study, which was to assess the temporal expression and localization of LMOD1 across the tissue during the regeneration process, we decided that cross-sectional analysis provided the most robust and reliable overview. Cross-sectional imaging effectively captures the spatial distribution of LMOD1 across multiple myofibers and their surrounding microenvironment, simultaneously assessing the whole cross-sectional area. By using this approach, we were able to evaluate the broader tissue architecture and cellular context, which was essential for understanding the dynamic changes occurring during regeneration. We were also able to investigate all myofibers of a muscle, and not only a small proportion, which we would analyze with longitudinal sections and isolated myofibers. Therefore, we continued using cross-sections for further analyses.

We fully agree with the reviewer that validating our in vitro SIRT1 findings in an in vivo context is an essential next step. To address this, we performed additional analyses on our existing regenerating muscle samples and incorporated new immunostainings for SIRT1 and PAX7 into the regeneration time-course (now shown in revised Figure 4I), providing further in vivo support for our proposed mechanism. We focused specifically on cross-sections collected at day 5 post-injury, a time point selected based on the peak in LMOD1 expression, to assess whether SIRT1 levels increase in parallel with LMOD1 during regeneration. Notably, SIRT1 abundance is elevated at day 5 post-injury, underscoring its involvement in early myogenic differentiation. This conclusion is further supported by the localization of SIRT1 within mononucleated cells and newly formed myofibers at this stage of regeneration.

Finally, we agree that further mechanistic studies in vivo would be highly valuable. While we were able to address SIRT1 dynamics in our regeneration model as suggested, an aged mouse cohort was unfortunately not available to us for this kind of study. Furthermore, more extensive in vivo experiments, such as those involving genetic manipulation, were beyond the scope of the current study, partly due to constraints related to animal welfare regulations and our approved experimental protocols.

Discussion:

Overall, the study emphasizes the importance of understanding the temporal dynamics of molecular players during myogenic differentiation and provides valuable proteomic data that will benefit the field. Future studies should explore whether LMOD1 modulates the nuclear-cytoplasmic shuttling of other transcription factors during muscle development and how these processes are mechanistically achieved. Investigating whether LMOD1 can be therapeutically targeted to enhance muscle regeneration in contexts such as exercise, aging, and disease will be critical for translational applications. Additionally, elucidating the interplay among LMOD1, LMOD2, and LMOD3 could uncover broader implications for actin cytoskeletal regulation in muscle biology.

We thank the reviewer for this excellent suggestion for future analyses. We have included these important considerations and future avenues in the Discussion of the revised manuscript:

“Our immunostaining data show that while LMOD1 is consistently cytoplasmic, its partner SIRT1 is only transiently localized in the cytoplasm. This suggests that their interaction is dynamically regulated. We hypothesize that the function of LMOD1 is determined by the changing availability of its binding partners during differentiation. During the initial phase, LMOD1 may primarily function to sequester SIRT1, a key regulator of myogenic genes. As differentiation proceeds, the increased expression of cytoskeletal components, such as its canonical partners TMODs and TPMs, likely shifts the function of LMOD1 towards its role in actin nucleation. This molecular switch, potentially driven by a change in the interactome of LMOD1, could then result in the release of SIRT1 from the cytoplasm. Such a mechanism may coordinate transcriptional regulation with cytoskeletal remodeling during myoblast differentiation.”

“Moreover, delineating the functional specialization and potential redundancy among leiomodin proteins represents an important next step. Our data indicate that LMOD1 primarily regulates early myogenic differentiation (Figure 3). In contrast, the lack of an early functional phenotype upon LMOD2 depletion, together with its upregulation at later stages (Figure S2A), suggests a temporal shift in regulatory control. Accordingly, a systematic comparative analysis of LMOD1, LMOD2, and LMOD3 will be required to elucidate their distinct roles in actin cytoskeleton regulation across the myogenic program, particularly with respect to myofibril maturation and maintenance.”

**Recommendations for the authors:**

**Reviewer #2 (Recommendations for the authors):**

Major Changes:

(1) In Vivo Data on SIRT1:

The inclusion of in vivo data on SIRT1 localization and expression would significantly strengthen the manuscript. Similar staining techniques used for LMOD1 could be applied to SIRT1. Additionally, imaging muscle specimens such as longitudinal sections or isolated myofibers would provide clearer insights into SIRT1's spatial distribution and improve upon the less convincing cross-sectional images currently presented (Figure 2).

We fully agree that providing in vivo data on SIRT1 localization and expression is a crucial step to support our in vitro findings. Following the reviewer's suggestion, we have performed new experiments on muscle regeneration samples using the analyses of cross-sections as done for the analysis of LMOD1 localization. Specifically, we performed immunostaining for SIRT1 on cross-sections from muscle samples collected at day 5 post-injury, a time point selected based on the observed peak in LMOD1 expression. These new data (now included in revised Figure 4I) allowed us to assess whether SIRT1 levels increase during regeneration in parallel with an increase in LMOD1 abundance.

Regarding the suggestion to use longitudinal sections or isolated myofibers, we agree that these preparations offer excellent answers for certain questions. For the primary goal of our study, to assess the temporal expression changes across the entire regenerating tissue at different time points, we found that cross-sections provided the most comprehensive and robust overview and therefore did not use longitudinal sections or isolated myofibers.

Performing additional animal experiments to obtain these specific preparations was beyond the scope of the current study and subject to constraints from our approved animal welfare protocols.

(2) Morphology of siLmod1 Cells:

The morphology of siLmod1-treated cells in vitro (Figure 3) raises concerns. Assessing cell viability or cell death in these experiments would help ensure that differences are not due to dead or unhealthy cells being quantified. There is also a notable discrepancy between the control panels in Figures 3C and 3H compared to the experimental conditions in 3F and 3K, particularly in terms of cell length and morphology. These inconsistencies should be addressed or clarified.

We acknowledge the visual discrepancies in cell morphology noted by the reviewer (e.g., between Figures 3C/3H and 3F/3K). These differences can be attributed to biological variability between primary myoblast cultures isolated from different mice. Such variability includes differences in myogenic potential and the fact that cells are not synchronized, leading to variations in differentiation efficiency, baseline morphology, and cell length across cultures (Cornelison 2008; Vaughan and Lamia 2019). To account for this, we decided to use n=6 biological replicates, i.e., primary myoblast cultures isolated from 6 different mice, for immunofluorescence analysis, ensuring robust quantitative data. Furthermore, we confirmed that this phenotype was not an artifact of culture conditions, as we consistently observed the same effect of Lmod1 knockdown independently of the passage number of the myoblasts or the donor mouse.

To address the concerns that morphological changes in siLmod1-treated cells might reflect cell death, we performed a TUNEL assay (transfection at day 1, analysis at day 3 of differentiation). This revealed no significant increase in TUNEL-positive (apoptotic) cells in siLmod1- (or siSirt1-) transfected samples versus siCtrl-transfected cells. These new data have been added to the revised manuscript as Supplementary Figure S2I. The TUNEL data indicate that the observed morphological changes upon knockdown of Lmod1 are not due to induced cell death. Supported by these results, our interpretation is that knockdown of Lmod1 impairs or arrests differentiation rather than causing cell death. Furthermore, our quantification of different cell populations showed shifts indicative of impaired differentiation (e.g., accumulation of cells at earlier stages) without exhibiting significant loss in cell numbers. For example, the numbers of myogenin+/MHC- and myogenin+/MHC+ cell populations, and differentiated myotubes, were not significantly reduced after transfection with siLmod1. A slight, not significant trend towards fewer non-proliferating myoblasts/reserve cells characterized by the expression of Myogenin-/MHC-Hoechst+ (Figure S2H) was noted. Overall, cells appeared to be 'stuck' in differentiation, consistent with the role of Lmod1 in impairing differentiation but not causing cell death. We have further clarified this aspect in the revised manuscript.

(3) LMOD1 and SIRT1 Interaction in Myogenic Cells:

Strengthening the connection between LMOD1 and SIRT1 within the myogenic system would enhance the manuscript. Could proximity ligation assays (PLA) be performed in myogenic cells, as was done in HEK293T cells? Additionally, investigating whether SIRT1 remains in the nucleus upon LMOD1 knockdown using siRNA would provide mechanistic insight into their interaction during myogenic differentiation.

We would like to clarify that the Proximity Ligation Assays (PLA) shown in Figure 4H were indeed performed in primary myoblasts, confirming the LMOD1-SIRT1 interaction directly in a myogenic context. We have modified the text to clarify that primary myoblasts were used for the PLA assays.

Minor Points:

(1) Was Lmod1 knockdown confirmed in vivo?

To target Lmod1 in Muscle Stem Cells (MuSCs) in vivo, we utilized self-delivering Accell siRNAs. This delivery system has been previously validated and shown to be highly effective for targeting MuSCs in regenerating muscle (Bentzinger et al., Cell Stem Cell, 2013).

While this is an established method for delivery, confirming knockdown specifically within the rare MuSC population is technically challenging using bulk tissue analysis, as the target signal is diluted by numerous other cell types.

Therefore, to ensure the efficacy of our specific siRNA, we performed in vitro validation. For the reviewers' interest, we add Author response image 2 showing the efficiency of the respective siRNAs:

**Author response image 2.** Knockdown efficiency of siRNAs targeting Lmod1 and Lmod2 following using the same self-delivering siRNA in proliferating primary myoblasts as used in in vivo experiments. Self-delivering Accell siRNA was added to primary myoblasts cultured in low serum media for 48 hours. Relative mRNA expression levels of *Lmod1* and *Lmod2* were measured after self-delivering Accell siRNA transfection targeting either *Lmod1* (siLmod1) or *Lmod2* (siLmod2). Expression levels were compared to control siRNA-transfected cells (siCtrl) and normalized to *Gapdh* expression.

Based on the documented efficacy of this delivery system from prior literature and our own validation of the specific siRNAs used here, we are confident in the knockdown efficiency of the respective siRNAs. We decided not to perform additional animal experiments due to animal welfare considerations.

(2) Some of the western blot bands do not appear to match the expected patterns for the tested proteins compared to controls (e.g., Figure S2J, S4C). Ensure that these are accurately labeled and include the entire membrane for transparency and reproducibility.

Regarding Figure S2J, we agree that the presentation could be confusing to the reader. The blot shows LMOD1 and LMOD2 knockdown, while the bar plot quantifies only the change in LMOD2 levels. We have now revised the figure legend to explicitly state this. We hope this makes the presentation of our data clearer.

For Figure S4C, we believe the concern about 'patterns' relates to loading variability. In this experiment, we manually counted the nuclei before lysis to ensure that each nuclear fraction started with an equal amount of material. We then loaded the cytoplasmic fractions in proportion to these counts. The purity of the fractions was additionally confirmed using nuclear (H4) and cytoplasmic (ALDOA) markers. As stated in the figure, the nuclear/cytoplasmic ratio of LMOD1 or SIRT1 was normalized across the entire lane of the Ponceau S staining, which we have now clarified in the relevant figure legends.

Finally, regarding transparency, the presented immunoblot images are representative crops, which is standard practice for clarity. We are committed to reproducibility and will provide full, uncropped scans of all blots in the final version of the manuscript, in line with eLife publishing guidelines.

(3) Figure S1B appears to reuse images from Figure 2D (rotated). Verify that this is acceptable for the journal's guidelines, and if necessary, provide additional justification or clarification.

We acknowledge that the image presented in Figure S1B was accidentally reused as a representative example in Figure 2D. To address this and prevent any potential redundancy or confusion, we have revised Figure S1B by replacing the duplicated image with a different, representative example from our dataset. The updated figure now contains unique image data, and we believe this revision fully resolves the concern.

(4) Ensure consistent scale bars across images, particularly in Figures 3C and 3H, where discrepancies might affect interpretation.

We thank the reviewer for pointing this out, we have now standardized all scale bars throughout the manuscript to ensure consistency. All immunofluorescence images of cultured cells (including Fig 3C and 3H) now have a 50 µm scale bar, and all tissue cross-sections have a 100 µm scale bar. This change has been implemented in the revised figures.

**Reviewer #3 (Public review):**

Summary:

In this manuscript, the investigators identified LMOD1 as one of a subset of cytoskeletal proteins whose levels increase in the early stages of myogenic differentiation. Lmod1 is understudied in striated muscle and in particular in myogenic differentiation. Thus, this is an important study. It is also a very thorough study - with perhaps even too much data presented. Importantly, the investigators observed that LMOD1 appears to be important for skeletal regeneration, and myogenic differentiation and that it interacts with SIRT1. Both primary myoblast differentiation and skeletal muscle regeneration were studied. Rescue experiments confirmed these observations: SIRT1 can rescue perturbations of myogenic differentiation as a result of LMOD1 knockdown.

Strengths:

Particular strengths include: important topic, the use of primary skeletal cultures, the use of both cell culture and in vivo approaches, careful biomarker analysis of primary mouse myoblast differentiation, the use of two methods to probe the function of the Lmod1/SIRT1 pathway via using depletion approaches and inhibitors, and generation of six independent myoblast cultures. Results support their conclusions.

We thank the reviewer for the positive assessment of our work and the helpful comments for improving our manuscript.

Weaknesses:

(1) Figure 1. Images of cells in Figure 1A are too small to be meaningful (especially in comparison to the other data presented in this figure). Perhaps the authors could make graphs smaller?

We have adjusted the size of the images across all figure panels to ensure better visibility and clarity. We hope these adjustments improve the presentation of the data.

(2) Line 148 "We found LMOD2 to be the most abundant Lmod in the whole skeletal muscle." This is confusing since most, if not all, prior studies have shown that Lmod3 is the predominant isoform in skeletal muscle. The two papers that are cited are incorrectly cited. Clarification to resolve this discrepancy is needed.

We acknowledge that LMOD2 and LMOD3 are predominantly expressed in skeletal and cardiac muscles (Tsukada et al. 2010; Nworu et al. 2015, www.proteinatlas.org) and LMOD3’s transcription is directly regulated by MRTF/SRF and MEF2 to coordinate sarcomeric assembly (Cenik et al. 2015). However, our statement refers specifically to the analysis of the proteomic datasets from freshly isolated MuSCs and four distinct skeletal muscles (G, S, TA, EDL) generated by Schüler et al. 2021. Crucially, LMOD3 was not detected in the quantitative mass spectrometry data for the EDL, G, S, or TA muscle samples analyzed in this specific study. In the context of this particular dataset, LMOD2 was the most highly abundant Leiomodin isoform detected in the whole skeletal muscle samples. This finding suggests a differential expression and function between LMOD isoforms depending on the muscle type and/or developmental/regenerative state. We have revised and corrected this clarification in the manuscript, including correcting the initial citations.

(3) Figure 2. Immunoflorescence (IF) panels are too small to be meaningful. Perhaps the graphs could be made smaller and more space allocated for the IF panels? This issue is apparent for just about all IF panels - they are simply too small to be meaningful. Additionally, in many of the immunofluorescence figures, the colors that were used make it difficult to discern the stained cellular structures. For example, in Figure S1, orange and purple are used - they do not stand out as well as other colors that are more commonly used.

We agree that the IF panels were too small for optimal interpretation and have adjusted them in Figure 2 and throughout the manuscript. Regarding the color choices, we appreciate the reviewer's comments. Our initial selection (e.g., orange and purple in Figure S1) was intended to enhance accessibility for individuals with common color vision deficiencies, including red-green color blindness. However, we acknowledge the reviewer's point that these combinations provided insufficient contrast for discerning cellular structures. Therefore, we have revised the color schemes to use green, red, and blue, which should offer improved contrast.

(4) There is huge variability in many experiments presented - as such, more samples appear to be required to allow for meaningful data to be obtained. For example, Figure S2. Many experimental groups, only have 3 samples - this is highly problematic - I would estimate that 5-6 would be the minimum.

We thank the reviewer for the comment regarding experimental variability and sample size. In our study, n=3 biological replicates, i.e., independent primary cell cultures obtained from different mice, were primarily used for immunoblots. We acknowledge that variability can be observed between distinct primary cell cultures due to factors such as inherent differences in myogenic potential, cell cycle state (as cells were not synchronized), and passage number. Importantly, despite this inter-sample variation, the investigated phenotypes showed consistent trends across biological replicates. Rather than increasing the number of replicates for immunoblots, we opted for validating our key findings using independent approaches with a higher number of replicates. For instance, qRT-PCR analyses (to confirm knockdown efficiency) and immunofluorescence analyses were mostly performed using five to six independent myoblast cultures (biological replicates).

(5) Ponceau S staining is often used as a loading control in this manuscript for western blots. The area/molecular weight range actually used should be specified. Not clear why in some experiments GAPDH staining is used, in other experiments Ponceau S staining is used, and in some, both are used. In some experiments, the variability of total protein loaded from lane to lane is disconcerting. For example, in Figure S4C there appears to be more than normal variability. Can the protein assay be redone and samples run again?

We have clarified in the relevant figure legends that Ponceau S normalization, when used, was based on the quantification of the entire lane. Our standard loading control is GAPDH. We used Ponceau S for normalization only when GAPDH was deemed unsuitable, e.g., in nuclear-cytoplasmic fractionation experiments where GAPDH is not present in all fractions.

Concerning the variability observed in Figure S4C, we manually counted the nuclei before lysis to ensure that each nuclear fraction started with an equal amount of material. We then loaded the cytoplasmic fractions in proportion to these counts. The purity of the fractions was additionally confirmed using nuclear (H4) and cytoplasmic (ALDOA) markers. The nuclear/cytoplasmic ratio of LMOD1 or SIRT1 was normalized across the entire lane of the Ponceau S staining, which we have now clarified in the relevant figure legends.

(6) Figure S3 - Lmod3 is included in the figure but no mention of it occurs in the title of the figure and/or legend.

We wish to clarify that the protein identified in Figure S3 is TMOD3 (Tropomodulin 3), not LMOD3. TMOD3 is a known pointed-end capping protein regulating the actin filament nucleation process together with LMODs (Fowler and Dominguez 2017; Boczkowska et al. 2015), so its presence in our dataset was expected and helps validate our results.

(7) Abstract, line 25. "overexpression accelerates and improves the formation of myotubes". This is a confusing sentence. How is it improving the formation? A little more information about how they are different than developing myotubes in normal/healthy muscles would be helpful.

We thank the reviewer for the comment. To clarify, we have revised the sentence in line 25 to "improves the initiation of myotube formation." This change reflects our observation that overexpression of LMOD1 leads to a more rapid onset of myotube formation, as evidenced by earlier expression of differentiation markers and accelerated fusion of myoblasts into myotubes compared to GFP overexpression myoblast cell line. These findings suggest that LMOD1 overexpression enhances the efficiency of the early stages of differentiation and fusion, thereby contributing to improved initiation of myotube formation.

(8) It is impossible from the IF figures presented to determine where Lmod1 localizes in the myocytes. Information on its subcellular localization is important. Does it localize with Lmod2 and Lmod3 at thin filament pointed ends?

Several publications suggest that LMODs are involved in actin nucleation and interact with TMODs at the thin filament pointed ends (Boczkowska et al. 2015; Fowler and Dominguez 2017; Fowler, Greenfield, and Moyer 2003; Tsukada et al. 2010; Rao, Madasu, and Dominguez 2014). We performed F-actin (Phalloidin) staining together with LMOD1 staining and observed possible co-localization (see Author response image 3). Specifically, we noted an accumulation of LMOD1 at the ends of myocytes, indicating that LMOD1 might play a role in the elongation and guidance of myotube differentiation. For the reviewer’s interest, we include Author response image 3 as it was not part of the original manuscript. While performing subcellular localization stainings, we added the F-actin/Phalloidin staining to explore potential interactions, but this aspect was not further investigated in the current study.

**Author response image 3.** Co-staining of LMOD1 and Phalloidin in differentiating myocytes. Example image showing immunofluorescence staining of LMOD1 (purple) and F-actin (green; Phalloidin) in differentiating primary myocytes. LMOD1 appears to accumulate at the ends of elongated myocytes and co-localizes with actin structures (highlighted in boxes), suggesting a potential role in myotube elongation and guidance during differentiation.

Our study focused on a distinct role for LMOD1, independent from its function in actin filament nucleation, and we therefore did not pursue further co-localization staining with LMOD2 or LMOD3. We recognize the potential importance of exploring these interactions and their relevance to thin filament organization in skeletal muscle. However, although this was beyond the scope of our current work, we will investigate this aspect in the future.

References

Boczkowska, Malgorzata, Grzegorz Rebowski, Elena Kremneva, Pekka Lappalainen, and Roberto Dominguez. 2015. “How Leiomodin and Tropomodulin Use a Common Fold for Different Actin Assembly Functions.” Nature Communications 6 (1): 8314.

Cenik, Bercin K., Ankit Garg, John R. McAnally, John M. Shelton, James A. Richardson, Rhonda Bassel-Duby, Eric N. Olson, and Ning Liu. 2015. “Severe Myopathy in Mice Lacking the MEF2/SRF-Dependent Gene Leiomodin-3.” The Journal of Clinical Investigation 125 (4): 1569–78.

Cornelison, D. D. W. 2008. “Context Matters: In Vivo and in Vitro Influences on Muscle Satellite Cell Activity.” Journal of Cellular Biochemistry 105 (3): 663–69.

Fowler, Velia M., and Roberto Dominguez. 2017. “Tropomodulins and Leiomodins: Actin Pointed End Caps and Nucleators in Muscles.” Biophysical Journal 112 (9): 1742–60.

Fowler, Velia M., Norma J. Greenfield, and Jeannette Moyer. 2003. “Tropomodulin Contains Two Actin Filament Pointed End-Capping Domains.” The Journal of Biological Chemistry 278 (41): 40000–9.

Liu, Ling, Tom H. Cheung, Gregory W. Charville, Bernadette Marie Ceniza Hurgo, Tripp Leavitt, Johnathan Shih, Anne Brunet, and Thomas A. Rando. 2013. “Chromatin Modifications as Determinants of Muscle Stem Cell Quiescence and Chronological Aging.” Cell Reports 4 (1): 189–204.

Lukjanenko, Laura, M. Juliane Jung, Nagabhooshan Hegde, Claire Perruisseau-Carrier, Eugenia Migliavacca, Michelle Rozo, Sonia Karaz, et al. 2016. “Loss of Fibronectin from the Aged Stem Cell Niche Affects the Regenerative Capacity of Skeletal Muscle in Mice.” Nature Medicine 22 (8): 897–905.

Nworu, Chinedu U., Robert Kraft, Daniel C. Schnurr, Carol C. Gregorio, and Paul A. Krieg. 2015. “Leiomodin 3 and Tropomodulin 4 Have Overlapping Functions during Skeletal Myofibrillogenesis.” Journal of Cell Science 128 (2): 239–50.

Rao, Jampani Nageswara, Yadaiah Madasu, and Roberto Dominguez. 2014. “Mechanism of Actin Filament Pointed-End Capping by Tropomodulin.” Science 345 (6195): 463–67.

Schüler, Svenja C., Joanna M. Kirkpatrick, Manuel Schmidt, Deolinda Santinha, Philipp Koch, Simone Di Sanzo, Emilio Cirri, Martin Hemberg, Alessandro Ori, and Julia von Maltzahn. 2021. “Extensive Remodeling of the Extracellular Matrix during Aging Contributes to Age-Dependent Impairments of Muscle Stem Cell Functionality.” Cell Reports 35 (10): 109223.

Tsukada, Takehiro, Christopher T. Pappas, Natalia Moroz, Parker B. Antin, Alla S. Kostyukova, and Carol C. Gregorio. 2010. “Leiomodin-2 Is an Antagonist of Tropomodulin-1 at the Pointed End of the Thin Filaments in Cardiac Muscle.” Journal of Cell Science 123 (Pt 18): 3136–45.

Vaughan, Megan, and Katja A. Lamia. 2019. “Isolation and Differentiation of Primary Myoblasts from Mouse Skeletal Muscle Explants.” Journal of Visualized Experiments: JoVE, no. 152 (October). https://doi.org/10.3791/60310.
